# Application of genomics-assisted breeding for generation of climate resilient crops: progress and prospects

**DOI:** 10.3389/fpls.2015.00563

**Published:** 2015-08-11

**Authors:** Chittaranjan Kole, Mehanathan Muthamilarasan, Robert Henry, David Edwards, Rishu Sharma, Michael Abberton, Jacqueline Batley, Alison Bentley, Michael Blakeney, John Bryant, Hongwei Cai, Mehmet Cakir, Leland J. Cseke, James Cockram, Antonio Costa de Oliveira, Ciro De Pace, Hannes Dempewolf, Shelby Ellison, Paul Gepts, Andy Greenland, Anthony Hall, Kiyosumi Hori, Stephen Hughes, Mike W. Humphreys, Massimo Iorizzo, Abdelbagi M. Ismail, Athole Marshall, Sean Mayes, Henry T. Nguyen, Francis C. Ogbonnaya, Rodomiro Ortiz, Andrew H. Paterson, Philipp W. Simon, Joe Tohme, Roberto Tuberosa, Babu Valliyodan, Rajeev K. Varshney, Stan D. Wullschleger, Masahiro Yano, Manoj Prasad

**Affiliations:** ^1^Bidhan Chandra Krishi ViswavidyalayaMohanpur, India; ^2^Department of Plant Molecular Genetics and Genomics, National Institute of Plant Genome ResearchNew Delhi, India; ^3^Queensland Alliance for Agriculture and Food Innovation, University of QueenslandSt Lucia, QLD, Australia; ^4^School of Agriculture and Food Sciences, University of QueenslandBrisbane, QLD, Australia; ^5^Department of Plant Pathology, Faculty of Agriculture, Bidhan Chandra Krishi ViswavidyalayaMohanpur, India; ^6^Genetic Resources Centre, International Institute of Tropical AgricultureIbadan, Nigeria; ^7^Centre for Integrated Legume Research, University of QueenslandBrisbane, QLD, Australia; ^8^The John Bingham Laboratory, National Institute of Agricultural BotanyCambridge, UK; ^9^Law School, University of Western AustraliaPerth, Australia; ^10^CLES, Hatherly Laboratories, University of ExeterExeter, UK; ^11^Forage Crop Research Institute, Japan Grassland Agriculture and Forage Seed AssociationNasushiobara, Japan; ^12^Department of Plant Genetics and Breeding, College of Agronomy and Biotechnology, China Agricultural UniversityBeijing, China; ^13^Faculty of Science and Engineering, School of Biological Sciences and Biotechnology, Murdoch UniversityMurdoch, WA, Australia; ^14^Department of Biological Sciences, The University of Alabama in HuntsvilleHuntsville, AL, USA; ^15^Plant Genomics and Breeding Center, Federal University of PelotasPelotas, Brazil; ^16^Department of Agriculture, Forests, Nature and Energy, University of TusciaViterbo, Italy; ^17^Global Crop Diversity Trust, Platz der Vereinten NationenBonn, Germany; ^18^Department of Horticulture, University of WisconsinMadison, WI, USA; ^19^Section of Crop and Ecosystem Sciences, Department of Plant Sciences, University of California, DavisDavis, CA, USA; ^20^Department of Botany and Plant Sciences, University of CaliforniaRiverside, Riverside, USA; ^21^Agrogenomics Research Center, National Institute of Agrobiological SciencesTsukuba, Japan; ^22^University of ExeterExeter, UK; ^23^Institute of Biological, Environmental and Rural Sciences, Aberystwyth UniversityWales, UK; ^24^Department of Horticulture, University of WisconsinMadison, WI, USA; ^25^International Rice Research InstituteManila, Philippines; ^26^Biotechnology and Crop Genetics, Crops for the FutureSemenyih, Malaysia; ^27^National Center for Soybean Biotechnology and Division of Plant Science, University of MissouriColumbia, MO, USA; ^28^Grains Research and Development CorporationKingston, ACT, Australia; ^29^Department of Plant Breeding, Swedish University of Agricultural SciencesSundvagen, Sweden; ^30^Plant Genome Mapping Laboratory, University of GeorgiaAthens, GA, USA; ^31^Department of Horticulture, USDA-ARS, University of WisconsinMadison, WI, USA; ^32^Agrobiodiversity and Biotechnology Project, Centro International de Agricultura TropicalCali, Columbia; ^33^Department of Agricultural SciencesBologna, Italy; ^34^Center of Excellence in Genomics, International Crops Research Institute for the Semi-Arid TropicsPatancheru, India; ^35^Oak Ridge National Laboratory, Environmental Sciences Division, Climate Change Science InstituteOak Ridge, TN, USA; ^36^National Agriculture and Food Research Organization, Institute of Crop ScienceTsukuba, Japan

**Keywords:** climate change, crop improvement, stress tolerance, breeding, genomics

## Abstract

Climate change affects agricultural productivity worldwide. Increased prices of food commodities are the initial indication of drastic edible yield loss, which is expected to increase further due to global warming. This situation has compelled plant scientists to develop climate change-resilient crops, which can withstand broad-spectrum stresses such as drought, heat, cold, salinity, flood, submergence and pests, thus helping to deliver increased productivity. Genomics appears to be a promising tool for deciphering the stress responsiveness of crop species with adaptation traits or in wild relatives toward identifying underlying genes, alleles or quantitative trait loci. Molecular breeding approaches have proven helpful in enhancing the stress adaptation of crop plants, and recent advances in high-throughput sequencing and phenotyping platforms have transformed molecular breeding to genomics-assisted breeding (GAB). In view of this, the present review elaborates the progress and prospects of GAB for improving climate change resilience in crops, which is likely to play an ever increasing role in the effort to ensure global food security.

## Introduction

Three major events in agricultural history, namely domestication, displacement of native crops by major crops, and intensification of agricultural production through the Green Revolution, have contributed significantly toward reduced genetic and trait diversity within major crop species (Pingali, 2012). Despite this decrease in crop diversity, global production of the major staple crops increased in the last century (Fischer et al., 2009). This increase in productivity has largely been driven by conventional plant breeding coupled with intensification and simplification of production systems. This includes selection for edible yield and adaptation, and against yield reducing factors such as susceptibility to pathogens to pests, as well as optimization of crop husbandry practices (through high inputs such as the use of fertilizers, herbicides, pesticides, and mechanization) to minimize the impact of environmental flux. However, selection under such “ideal,” high-input environments has led to the loss of certain genes which are responsible for efficiency or adaptation to stress(es) (Brown, 2003). This situation presents three potential challenges: (i) to modify the selection criteria to focus on efficiency or adaptation to stress(es) rather than total edible yield, (ii) to ensure the presence and efficiency of stress-tolerance genes and their exploitability in elite material and wider breeding germplasm, and (iii) to expand the use of minor crops, which may possess better nutritional qualities, environmental sustainability or resilience and require lower inputs than major crops.

At present, global agriculture is facing a serious threat from climate change, which is predicted to result in reduced productivity. Increasing food prices and greater global food insecurity are the outcomes of decreased productivity (FAO, 2014) and this scenario, if it persists, would lead to further increase in food prices, and could lead to social unrest and famine in certain instances. Climate change will affect food supply unless actions are taken to increase the resilience of crops: projections have shown a drastic decrease in the production of major cereals by 2020, including 9% for maize, 11% for rice, and 14% for wheat (Hisas, 2011). Global warming, changes in rainfall patterns and other extreme weather events may mostly contribute to this disaster, and the changing pattern of climate would result in increased attack of pathogens and pests. Moreover, the elevated CO_2_ levels are predicted to reduce the nutritional quality of many crops, while some crops may become toxic due to changes in the chemical composition of their tissues (Dwivedi et al., 2013).

Therefore, increasing the resilience of crops to climate change represents a critical component towards ensuring food and nutritional security, which could be achieved through genetic engineering-based approaches or molecular breeding strategies. Genetic engineering allows direct transfer of beneficial gene(s) or manipulation of existing gene(s) in the crop of interest for generating expected phenotype(s), whereas breeding approaches involve the improvement of germplasm through introduction of novel alleles into target crops by breeding. Since genetic modification remains controversial in a number of countries (though it serves as an invaluable tool in tailoring modifications to produce alleles and phenotypes beyond the range available through exploitation of existing genetic variation), molecular breeding could offer an easy-to-accept approach for crop improvement.

## Potential of genomics-assisted breeding in producing climate resilient crops

Genomics offers tools to address the challenge of increasing food yield, quality and stability of production through advanced breeding techniques. Application of DNA markers to facilitate marker-aided selection (MAS) for crop improvement have proved successful in crossbreeding. Advances in plant genomics provide further means to improve the understandings of crop diversity at species and gene levels, and offer DNA markers to accelerate the pace of genetic improvement (Muthamilarasan et al., 2013, 2014). A genomics-led breeding strategy for new cultivars for the development of new cultivars that are “climate change ready” (Varshney et al., 2005) commences by defining the stress(es) that will likely affect crop production and productivity under certain climate change scenarios. Data from multi-environment testing provide an opportunity for modeling “stress-impacts” on crops and target populations of environments. Plant breeders and genebank curators will search for morphological and physiological traits in available germplasm that could enhance crop adaptation under such climate variability. In this regard, crop physiology may help define the ideotypes to be pursued for enhancing such adaptation. Moreover, the use of geographic information systems and passport data can allow identification of accessions from stress-prone environments, whereas the available characterization, including DNA fingerprinting, and evaluation data as well as mapping of desired genes or quantitative trait loci (QTL) will assist in selecting promising accessions for further screening against specific stress(es). Similarly, precise phenotypic assessments and appropriate biometric analysis will assist in identifying unique responses of a set of genotypes in a given physiological stage influenced by variation of weather patterns. This information will be further used in genomics-aided breeding approaches such as genome-wide selection of promising germplasm for further use in crop breeding aiming at both population improvement and cultivar release.

Genetic mapping and QTL analysis, via bi-parental or association mapping (AM) populations, have accelerated the dissection of genetic control of agricultural traits, potentially allowing MAS, QTL, and AM studies or direct calculation and genomic selection (GS) of high value genotypes to be made in the context of breeding programs (Kulwal et al., 2011). Until recently, AM and GS were hampered by the need for very high marker density coverage of the genome. Advancement of next-generation sequencing (NGS) methods has facilitated the development of large numbers of genetic markers, such as single nucleotide polymorphisms (SNP), insertion-deletions (InDels), etc. even in relatively research-neglected crop species. Discovery of novel genes/alleles for any given trait could be then performed through genotyping-by-sequencing (GBS) approaches. Similarly, genome-wide association studies (GWAS) could be used to identify the genomic regions governing traits of interest by performing statistical associations between DNA polymorphisms and trait variations in diverse collection of germplasms that are genotyped and phenotyped for traits of interest. NGS coupled with GWAS increases the mapping resolution for precise location of genes/alleles/QTL (Ma et al., 2012; Liu et al., 2013; Varshney et al., 2014).

During the course of evolution, nature has evolved new genes, and shuffled and selected these genes in a wide range of environments to produce the diversity evidenced in wild species. In contrast, the selection and domestication of crops by humans is relatively recent, having occurred over the last 10,000 years. During the domestication and breeding process, there has been a significant reduction of genetic diversity in major crops, alongside a selection for yield under highly managed agricultural environments. Currently, breeders are shuffling relatively few alleles to produce enhanced combinations that provide increased yield and other attractive agronomic characteristics. In many large-genome crop species, even this reshuffling process is limited by restricted recombination patterns within the species, leading to the consistent inheritance of blocks of genes, raising issues of linkage drag and fixed linkage blocks, which may not contain the best possible combination of alleles. Breaking down these linkage constraints will allow breeders to access novel allele/gene combinations from within their current elite parents. The need to evaluate the genetics of the processes that allow genes to be recombined between parental genotypes in crops is a critical requisite. Genomics possesses the potential to increase the diversity of alleles available to breeders through mining the gene pools of crop wild relatives (CWRs). Genomics tools also enable rapid identification and selection of novel beneficial genes and their controlled incorporation into novel germplasm. In the genomics era, this technology will be used to safeguard the future through improved food security. Taken together, the application of genomics for crop germplasm enhancement offers the greatest potential to increase food production in the coming decades. With continued rapid advances in genome technologies, the application of genomics to identify and transfer valuable agronomic genes from allied genepools and crop relatives to elite crops will increase in pace and assist in meeting the challenge of global food security.

## Genomics of climate resilience in major crops

The following section summarizes the state of knowledge of the genetic blueprints of many leading crops, together with information about breeding needs and priorities related to climate resilience. Genomic tools and resources are widely available and being employed in most of these species and will soon be ubiquitous, aiding “MAS” strategies that can be successful even based only on phenotypic information. Knowledge of gene functions is less consistent, leveraging to varying degrees the accumulated information from plant model species. However, even in model crops, the exact functions of most genes remain unknown, and exploring the variation conferred during angiosperm diversification represents an opportunity to identify a host of solutions to agricultural challenges.

## Cereals

Cereals are a staple to billions and their production is increasingly threatened by the recent changes in weather patterns due to global warming, particularly in less-developed countries where the consequences of changing climate have devastating socio-economic impact. Reaching a level of cereal production sufficient to sustain an adequate level of global food security will require the effective integration of crossbreeding with “omics” approaches that allow dissecting and more effectively manipulating the genetic make-up of adaptation to abiotic stresses (Langridge and Fleury, 2011). In the past decade, genomics-based approaches have been extensively deployed to dissect the genetic make-up of abiotic stress adaptation and given the quantitative nature of abiotic stress tolerance, QTL have been the main target of research to identify the genetic loci regulating the adaptive response of cereal crops to unfavorable environmental conditions. This includes drought-adaptive traits (Serraj et al., 2009; Tuberosa, 2012), root architecture (Wasson et al., 2012; Uga et al., 2013; Lynch et al., 2014), accumulation of water-soluble carbohydrates and their partitioning to storage organs (Landi et al., 2005; Salem et al., 2007; Snape et al., 2007; Rebetzke et al., 2008), abscisic acid concentration (Rebetzke et al., 2008; Rehman et al., 2011), stay-green (Yang et al., 2007; Borrell et al., 2014), canopy temperature (Lopes et al., 2014), and carbon isotope discrimination (Δ^13^C) (Pinto et al., 2010).

Global warming is intimately associated with an increase in temperature that accelerates leaf senescence, disrupts starch accumulation and curtails yield, particularly when combined with drought. In wheat, a major QTL located on chromosome 4A explained 27 and 17% of phenotypic variance for reduction in yield under drought and heat stress, respectively (Pinto et al., 2010). The same study also identified common QTL for drought and heat stress adaptation on chromosomes 1B, 2B, 3B, 4B, and 7A. Yield QTL were shown to be associated with components of other traits, supporting the prospects for dissecting crop performance under abiotic stress conditions into physiological and genetic components in order to facilitate a strategic approach to breeding (Reynolds and Tuberosa, 2008). Additional QTL with concurrent effects under both heat and drought conditions have been described by Wang et al. (2012) and Paliwal et al. (2012).

In rice, the result of a study based on 227 intensively managed irrigated farms forecast a net negative impact on yield from the warming expected in the coming decades, and clearly show that diurnal temperature variation must be considered when investigating the impact of climate change (Welch et al., 2010). Higher temperatures are speculated to reduce rice grain yields through two main pathways: (i) high maximum temperatures that in combination with high humidity cause spikelet sterility, and (ii) increased nighttime temperatures, which may reduce assimilate accumulation (Wassmann and Jagadish, 2009).

Flooding is one of the abiotic stresses, whose frequency and intensity is increasing due to global warming and changes in rainfall patterns. Therefore, it is important to produce cereal crops with the ability to withstand the anoxic conditions associated with waterlogging and/or extended submergence. Among cereals, rice is more prone to submergence stress, which periodically affects approximately 15 million hectares of rain-fed lowland areas in Asia to cause annual losses in excess of US $1 billion (Mackill et al., 2012). In rice, the *Sub1* QTL accounts for a major portion of variability for survival under prolonged submergence. Positional cloning of *Sub1* has revealed a cluster of three putative ethylene response factors (ERFs), namely *Sub1A, Sub1B*, and *Sub1C*. Further work unequivocally assigned the functional polymorphism to *Sub1A* (Xu et al., 2006). Following the identification of *Sub1A* QTL, marker-aided backcrossing (MABC) was used to efficiently convert submergence-susceptible rice cultivars into tolerant cultivars in only three backcross generations. Accordingly, DNA markers were developed for introgressing *Sub1* into six popular cultivars to meet the needs of farmers in flood-prone regions (Bailey-Serres et al., 2010). This clearly demonstrates the effectiveness of MAS for introgressing agronomically beneficial QTL alleles into elite material. The success of this work is largely due to the major effect of *Sub1* QTL and the stability of its effect in different genetic backgrounds under submergence conditions. In maize, Mano et al. (2005a) identified QTL for adventitious root formation at the soil surface, one of the most important adaptations to soil waterlogging, which can severely impair root growth at an early stage, thus reducing the capacity of the plant to extract soil moisture at a later stage when water shortage is more likely to occur. Several QTL for adventitious root formation have been mapped, and a major QTL was mapped on chromosome 8 (Mano et al., 2005b).

Salinity is also an impact of global climate change, which affects over 20% of the world's agricultural soils and thereby affecting cultivation. In durum wheat (genome AABB), two major QTL have been shown to control Na^+^ accumulation in shoot via Na^+^ exclusion (James et al., 2006). Both exclusion genes represent introgressions from an accession of *Triticum monococcum* (genome AA). Remarkably, under standard conditions, durum wheat containing the salinity tolerant allele at *TmHKT1;5-A*, which is one of the two salt-tolerance loci showed the phenotype similar to durum wheat that lacked the beneficial allele at this locus. But under saline conditions, it outperformed its durum wheat parent, with increased yields of up to 25% (Munns et al., 2012). In barley, evaluation of a mapping population derived from a cross between a wild barley (*Hordeum vulgare* ssp. *spontaneum*) accession and cultivated barley (*H. vulgare*) allowed the identification of a major QTL capable of limiting Na^+^ accumulation in the shoots under saline conditions (Shavrukov et al., 2010). In rice, several QTL for salinity tolerance have been identified (Wang et al., 2012) indicating that pyramiding by marker-assisted selection (MAS) of QTL can be applied to enhance salt tolerance of rice.

## Oilseeds and pulses

Oilseeds and pulses are major food crops, known for their unique protein and oil rich characteristics. Major biotic and abiotic stresses are the most serious production constraint for global oilseed and pulse production, and are predicted to worsen with anticipated climate change. Among the oilseeds, soybean has the highest protein content (40%) and the second highest oil content (20%). In spite of this importance, efforts are yet to be invested toward improving stress tolerance and other traits in soybean. *Phaseolus* beans are an essential part of the human diet and are a source of proteins, vitamins, and minerals (Gepts and Aragão, 2008). Of the five domesticated *Phaseolus* species, common bean (*P. vulgaris* L.) is the most economically important bean. Genetic studies and cultivar breeding in *P. vulgaris* have shown that heat and drought tolerance are under complex genetic control, although a single instance of a major gene has also been observed (Schneider et al., 1997; Asfaw et al., 2012). Selection of lines with improved drought adaptation has also been successful (Singh, 2007; Beebe et al., 2008; Urrea et al., 2009). Development of MAS methodology for drought adaptation has been initiated (Schneider et al., 1997; Asfaw et al., 2012) with the assistance of genomic resources developed through whole-genome sequencing of Andean (accession: G19833) (Schmutz et al., 2014) and Mesoamerican (accessions: BAT93, OAC Rex) bean genomes, and a bean breeder's genome toolbox and database (http://phaseolusgenes.bioinformatics.ucdavis.edu/).

In case of chickpea and pigeonpea, several abiotic and biotic stresses pose a threat to high and stable grain yields. To overcome these production constraints and meet the growing demand for these crops, efforts at national and international levels have led to the development of large-scale genetic and genomic resources (Varshney et al., 2013a). These resources have been used to understand the existing genetic diversity and exploit it in breeding programs. In chickpea, several intra- and inter-specific genetic maps have been developed (Gaur et al., 2011; Gujaria et al., 2011; Thudi et al., 2011; Hiremath et al., 2012) and genomic regions responsible for different biotic stresses (Anbessa et al., 2009; Kottapalli et al., 2009; Anuradha et al., 2011), abiotic stress (Rehman et al., 2011; Vadez et al., 2012) and agronomic traits (Cobos et al., 2009; Rehman et al., 2011; Bajaj et al., 2014, 2015; Das et al., 2015; Kujur et al., 2015a,b) have been reported. In pigeonpea, more than 3000 SSR markers (Saxena et al., 2010; Bohra et al., 2011; Dutta et al., 2011), ESTs (Raju et al., 2010), 454/FLX transcript reads (Dubey et al., 2011; Dutta et al., 2011), transcriptome assemblies (Dubey et al., 2011; Kudapa et al., 2012), and SNPs (Saxena, 2008) have been developed for their use in genomics-assisted breeding for crop improvement.

The draft genome sequence of both Kabuli (http://www.icrisat.org/gt-bt/ICGGC/GenomeSequencing.htm) and Desi (http://www.nipgr.res.in/CGWR/home.php) chickpeas have recently been published (Jain et al., 2013; Varshney et al., 2013b). Similarly, International Initiative on Pigeonpea Genomics (IIPG, http://www.icrisat.org/gt-bt/iipg/Home.html) released the draft genome of pigeonpea (Varshney et al., 2012). These sequence data of chickpea and pigeonpea will assist in enhancing their crop productivity and lead to conserving food security in arid and semi-arid environments. Further, attempts have been made toward improvement of oilseed crops such as peanut (or groundnut) using genomics-assisted breeding. Large-scale genomic resources were developed during recent years to facilitate molecular breeding in peanut and QTL have been identified for stress adaptation related traits (Varshney et al., 2009; Gautami et al., 2012), rust and late leaf spot resistance (Khedikar et al., 2010; Sujay et al., 2012), and oil quality (Sarvamangala et al., 2011).

## Millets

Millets are small-grained graminaceous crops, well-known for their water-use efficiency, excellent nutrient content, adaptation to a range of ecological conditions and ability to flourish in nutrient-poor soils. Foxtail millet, proso millet, pearl millet, barnyard millet, finger millet, and kodo millet are few notable millet crops cultivated worldwide and of these, foxtail millet is considered as a C_4_ crop model for studying the biology of other millets and biofuel grasses (Lata et al., 2013; Muthamilarasan and Prasad, 2015). Therefore, the Beijing Genomics Institute, China and the US Department of Energy–Joint Genome Institute have sequenced the foxtail millet genome (Bennetzen et al., 2012; Zhang et al., 2012). As foxtail millet serves as a rich source of genes, alleles, or QTL for genetic improvement of major cereals and bioenergy grasses, large-scale genomic resources were developed including simple sequence repeats (SSRs) (Pandey et al., 2013; Zhang et al., 2014b), intron length polymorphisms (Muthamilarasan et al., 2014), eSSRs (Kumari et al., 2013), miRNA-based markers (Yadav et al., 2014), and transposable-elements based markers (Yadav et al., 2015). Moreover, open access online databases such as foxtail millet marker database (FmMDb) (Suresh et al., 2013), foxtail millet miRNA database (FmMiRNADb) (Khan et al., 2014) and foxtail millet transposable elements-based marker database (FmTEMDb) (Yadav et al., 2015) have been constructed. In addition to development of these markers, their utility in population genetics, association mapping, comparative genomics and genomics-assisted breeding have also been demonstrated (Muthamilarasan and Prasad, 2015). An allele-specific marker developed from an SNP in *SiDREB2* gene linked to drought tolerance in foxtail millet (Lata et al., 2011) is being used in allele mining and MAS for drought tolerance (Lata and Prasad, 2012, 2013).

In pearl millet, three major QTL for grain yield with low QTL × environment interactions were identified across a range of post-flowering moisture environments (Bidinger et al., 2007). One of these major QTL accounted for up to 32% of the phenotypic variance of grain yield under drought. The effects of this QTL were validated in two independent MABC programs in which 30% improvement in general combining ability for grain yield expected from this QTL under terminal drought stress was recovered in introgression lines, based on the information provided by the markers flanking the QTL (Yadav et al., 2011). Compared to other crops, research on millets is at initial stage. Being predominantly climate resilient crops, millets could serve as valuable source of novel genes, alleles and QTL for stress tolerance, which needs to be identified and characterized. The close phylogenetic relationships between millets and other cereals could enable the introgression of novel alleles, genes or QTL identified in millets for better agronomic traits into other cereals toward ensuring food security under changing climate.

## Forest and fruit tree crops

Clones of trees, namely populus, pinus, abies, and eucalyptus are used in afforestation, as they are dedicated to the production of wood and other wood-derived products. Therefore, it is imperative to develop climate-change resilient clones or populations of these forest trees. Several procedures have been developed for high-throughput DNA genotyping and genome-wide marker identification in forest trees. The genome complexity reduction DArT (Alves-Freitas et al., 2011) and whole-exome capture using in-solution target enrichment (Neves et al., 2011) have been tested successfully for genome-wide marker identification needed for GS in *Pinus taeda*. Considering the importance of genome sequence for development of genetic markers, Conifer Genome Project (http://www.pinegenome.org/cgp/) has been launched with an aim of promoting advance genome research in loblolly pine (*P. taeda*; 21.7 Gbp/1C; *n* = 12), white pines (*Pinus* subgenus *strobus*; 25.1 Gbp/1C; *n* = 12), as well as *Sequoia sempervirens* (31.4 Gbp/1C; *n* = 3x = 33) and Douglas-fir (*Pseudotsuga menziesii*; 18.6 Gbp/1C; *n* = 13). An extensive genetic resources and gene catalog was developed for *P. taeda* and *Picea glauca* (white spruce; 19.7 Gbp/1C; *n* = 12) (http://www.pinegenome.org/cgp/). The GENOAK project (http://urgi.versailles.inra.fr/Projects/GenOak) aims to establish a high quality reference genome sequence for pedunculate oak (*Quercus robur*; 905 Mbp/1C; *n* = 12). The *Eucalyptus grandis* (640 Mbp/1C, *n* = 11) genome has been deciphered (http://phytozome.jgi.doe.gov/pz/portal.html#!info?alias=Org_Egrandis) and will benefit agro-foresters utilizing this fast-growing hardwood tree to support industries based on Eucalypt fiber and hardwood products, and the production of Eucalypt feedstock for cellulosic biofuels. Importantly, this would assist in accelerating forest tree breeding for fast response to the need of adapted populations facing environmental modifications induced by climate change.

Fruit trees are also as important as the pulse and cereal crops and climate-change resilient clones or populations of fruit tree crops are necessary to maintain the source of nutrients that help the daily intake of healthy food ingredients. Genomics-based breeding approaches, along with bioinformatics capability and other omics resources will be the essential components of perennial fruit crop breeding and particularly, to adapt their cropping to combat or mitigate climate change effects. Genome sequencing and annotation projects include perennial fruit crops such as apple (Velasco et al., 2010), grape (Velasco et al., 2007), banana (D'Hont et al., 2012), cocoa (Argout et al., 2011), peach (Ahmad et al., 2011), and sweet orange (Xu et al., 2013). The advances in genome sequencing, along with high-resolution genetic mapping and precise phenotyping will accelerate the discovery of functional alleles and allelic variations that are associated with traits of interest for perennial fruit crop breeding. However, very less progress has been made in this aspect and particularly, enhancing climate resilience needs more attention. For achieving this, genetics and genomics methodologies could provide the toolbox for identifying genomic regions associated with the desired phenotype, and assist the selection from the wild genetic resources of the parental plants that will be intercrossed to provide the progenies for commencing breeding procedures for recurrent selection.

## Genomics-assisted breeding strategies for climate resilient traits

**Figure 1:**
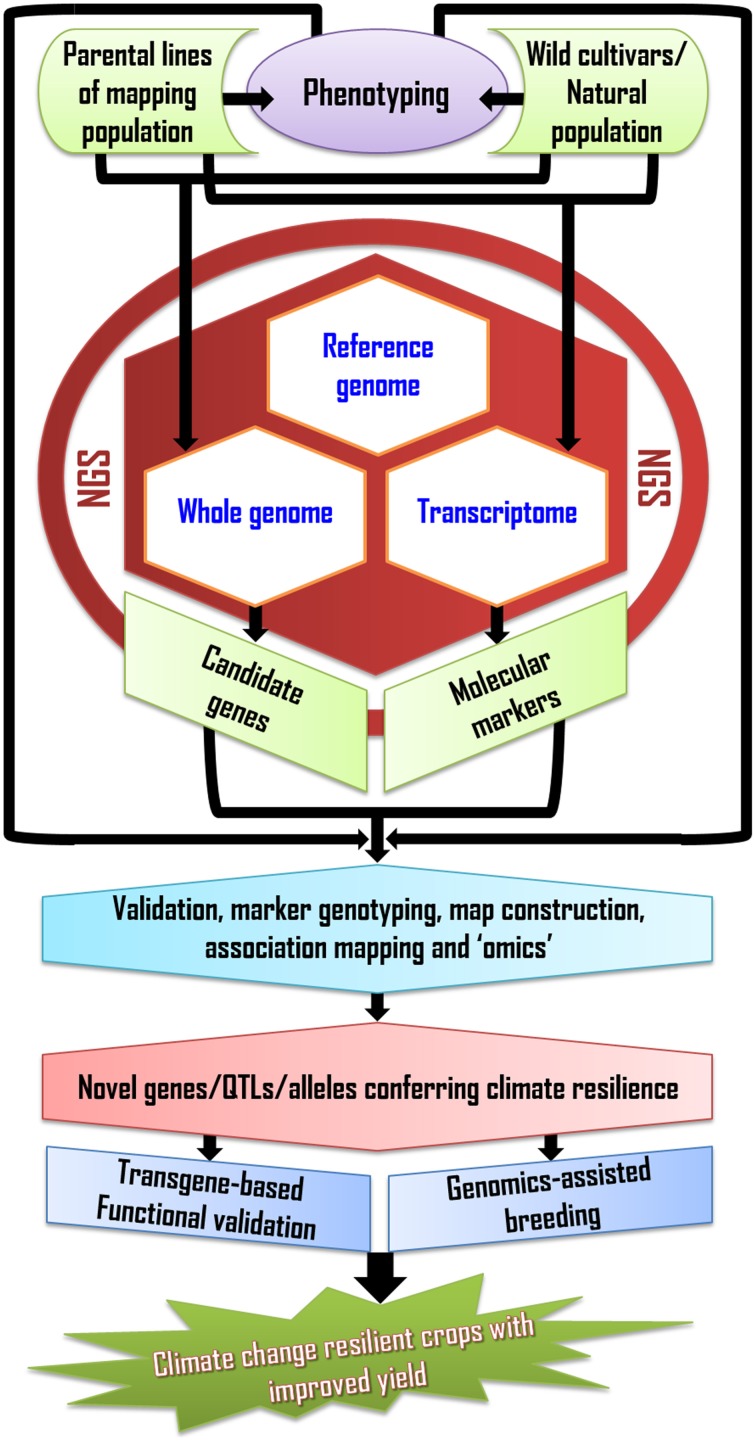
**Flow-chart demonstrating the steps involved in generating climate resilient crops using genomics and next-generation sequencing technology**.

Genomics-based approaches and NGS have ushered in sequence-based breeding strategies that will expedite the dissection and cloning of the loci controlling abiotic stress tolerance, while providing unparalleled opportunities to tap into wild relatives of crops, hence expanding the reservoir of genetic diversity available to breeders (Tuberosa et al., 2011; Edwards et al., 2013) (Figure 1). In view of the genetic complexity of yield, particularly under drought and other abiotic stresses, GS will provide the most powerful approach to raise the yield potential to the levels required to meet the fast-increasing global demand in cereal grain. However, MAS will remain a valid option for major loci or QTL, while QTL cloning will become a more routine activity facilitated through increased utilization of high-throughput, accurate phenotyping (Araus and Cairns, 2014), sequencing (Imelfort et al., 2009; Edwards and Wang, 2012), and identification of suitable candidate genes through “omics” profiling (Gupta et al., 2013). Cloned QTL will provide novel opportunities for genetic engineering for abiotic stress tolerance and for a more targeted search for novel alleles in wild germplasm (Salvi et al., 2007). Even with the application of advanced genomics technologies, mitigating the negative effects of climate change on crop productivity will remain a daunting undertaking. This requires a multidisciplinary and integrated approach, which will eventually allow plant breeders to more effectively select crops that are more resilient to climate change and ensure a sufficient level of food security for the decades to come.

### Flowering time and drought adaptation

Temperature influences crop development in concert with additional floral pathways such as day-length (photoperiod), which collectively control floral transition through interconnected genetic pathways. Global warming will result in increased ambient temperature with unchanged photoperiods at given latitudes. Annual plants generally respond to increased temperatures with accelerated growth and development, having shortened lifecycles, less opportunity for photosynthesis (Reynolds et al., 2010), a shorter reproductive phase and lower yield potential (Ainsworth and Ort, 2010). There is also an increased risk of damage to reproductive tissue caused by the coincidence of high temperatures and sensitive developmental stages. Therefore, detailed knowledge of the interplay between genetic control of flowering, allelic variants, epistatic interactions, and phenotypic variations in varied growth conditions is necessary in order to identify breeding targets for climate change scenarios.

There are increasing number of germplasm resources including precise near isogenic lines (NILs) (Bentley et al., 2011, 2013) as well as next-generation populations such as multi-founder populations (e.g., multi-parent advanced generation intercross populations), which have been developed in wheat (Mackay et al., 2014) and other crops to facilitate further research and validation of climate-smart crops. New variation incorporated into elite backgrounds from landraces, ancestral or wild crop relatives (e.g., www.wheatisp.org) also offers potential for discovery of functional variation for manipulating flowering time to suit future climate permutations. However, initial work should focus on understanding the effect of flowering time on yield potential across environments and environmental stresses. Identifying the potential utility of loci of minor effect and/or which affect various stages of reproductive development could offer the ability to shorten or lengthen various phases of the flowering process, thereby enabling fine-tuning of flowering to suit particular regional climatic conditions, and to adapt to any changes in these conditions.

In case of drought tolerance, multi-disciplinary research is underway to improve plants' response to drought and water-use efficiency. With the advent of molecular breeding, QTL identification and QTL use in breeding programs assist in developing new cultivars with improved drought tolerance. In maize, extensive work has been carried out to investigate the role of root in mitigating the negative effects of drought. QTL for root traits have been described in a number of maize populations (Ruta et al., 2010; Hund et al., 2011; Tuberosa et al., 2011) in which some QTL showed a concurrent effect on grain yield performance under drought (Landi et al., 2005). Recently, Syngenta and Pioneer-DuPont deployed proprietary genomics-assisted approaches to select drought-tolerant maize hybrids (Agrisure Artesian™ and AQUAmax™, respectively) (Cooper et al., 2014). The superior performance of these maize hybrids in the severe drought that plagued the US Corn Belt in summer 2012 underlines their validity under dry soil conditions (Cooper et al., 2014). In wheat, yield QTL were shown to be associated with components of other traits, supporting the prospects for dissecting crop performance under abiotic stress conditions into its physiological and genetic components in order to facilitate a strategic approach to breeding (Reynolds and Tuberosa, 2008). At least 15 different populations have been used to map drought adaptation in rice and four regions were identified as key for yield or yield components under stress, and drought-tolerant component traits were identified across populations with interval lengths of 35–64 cm (Kamoshita et al., 2008). The first region (on chromosome 1) was associated with grain yield drought-resistance traits, plant type traits (Zhang et al., 2001a), and QTL for cell-membrane stability (Tripathy et al., 2000) and osmotic adjustment (Lilley et al., 1996), and root traits (Robin et al., 2003). Second genomic region on chromosome 4 was rich in root trait QTL (Hemamalini et al., 2000; Zheng et al., 2000; Zhang et al., 2001b; Kamoshita et al., 2002; Nguyen et al., 2004; Boopathi et al., 2005) under well-watered and drought conditions. The third region located on chromosome 8 contained QTL for plant water status, grain yield, cell membrane stability, osmotic adjustment, rate of non-stomatal water loss and deep and thick root traits (Hemamalini et al., 2000; Zheng et al., 2000; Zhang et al., 2001b; Nguyen et al., 2004; Boopathi et al., 2005). The fourth important region for drought was located in chromosome 9, which was characterized by QTL for root traits, cell membrane stability, plant water status, leaf rolling and leaf drying, biomass, number of grains per panicle, relative spikelet fertility and delay in flowering time (Hemamalini et al., 2000; Tripathy et al., 2000; Zhang et al., 2001a; Kamoshita et al., 2002; Price et al., 2002a,b; Babu et al., 2003; Courtois et al., 2003; Robin et al., 2003; Zheng et al., 2003; Lafitte et al., 2004; Lanceras et al., 2004; Nguyen et al., 2004; Boopathi et al., 2005; Gomez et al., 2005; Li et al., 2005; Xu et al., 2005; Yue et al., 2006; Jearakongman, 2005). Since these four regions are consistently reported to be associated with drought response and stood above the average, these regions should be part of marker assisted breeding program for drought tolerance in rice.

## Cold and heat stress tolerance

Tolerance to freezing temperatures is the most important component for winter survival, but also of considerable importance is the capability to withstand combinations of stresses due to desiccation, wind, ice-encasement, heaving, low light, snow cover, winter pathogens, and fluctuating temperatures. Resistance to desiccation through the maintenance of cell membrane integrity and retention of cellular water is essential, and it is unsurprising that the same genetic response to the onset of freezing temperatures is often observed with drought or salinity stress (Yue et al., 2006). Indeed, cold acclimation (CA) can frequently improve adaptation to a mild drought stress and *vice versa* (Seki et al., 2002). Major genes or gene clusters involved in the control of frost and drought adaptation are located on a region of the long arm of Triticeae group 5 chromosomes. Traits such as winter hardiness (Thomas and James, 1993), vernalization response and frost tolerance (Galiba et al., 1995; Hayes et al., 1998), cold- and drought-induced ABA production (Laurie et al., 1995), and osmotic stress-tolerance (Galiba et al., 1993), have all been mapped to this region. Across the grasses and cereals, this chromosome region has been a major focus for genome research and for plant breeding. It may well be as consequence of climate change from the perspective of future crop design that in many locations where winter temperatures are on the increase and favoring continued plant growth, and where this is accompanied by a decrease in winter rainfall, that unseasonal winter droughts will ensue, which will require a new breeding strategy for common stress tolerance to both stress factors.

The C-repeat binding factor (CBF) genes are key regulators of the expression of COR (cold regulated genes), which are conserved among diverse plant lineages such as eudicots and monocots. The CBF transcription factors recognize the cis-acting CRT/DRE (C-repeat/dehydration responsive element) element in the regulatory regions of COR genes (Stockinger et al., 1997). Twenty CBF genes have been identified in barley (*H. vulgare*), of which 11 are found in two tight tandem clusters on the long arm of chromosome 5H in the same region as the *Fr-H2* frost tolerance locus (Skinner et al., 2006; Francia et al., 2007). An orthologous genomic region in *T. monococcum* contains similar CBF gene clusters located at the *Fr-A*^*m*^*2* frost tolerance QTL (Vagujfalvi et al., 2003; Miller et al., 2006). Studies of the organization of CBF cluster in barley and wheat have shown that the number of CBF genes at *Fr-H2/Fr-A1* locus may vary among cultivars with winter forms having a higher copy number of some CBFs (Francia et al., 2007; Knox et al., 2010). The co-segregation of CBF gene clusters with barley *Fr-H2* and wheat *Fr-A*^*m*^*2* frost tolerance loci, their role in cold acclimation (Stockinger et al., 1997), and the association of transcript levels of CBF genes with frost tolerance loci (Vagujfalvi et al., 2003) make them obvious candidates for one of the two major frost tolerance QTL on Triticeae group 5 chromosomes. The locations of two frost tolerance/winter survival QTL on the chromosome 5F of forage grass *Festuca pratensis* correspond most likely to the *Fr-A1* and *Fr-A2* loci on wheat homoeologous group 5A chromosomes. One of these QTL (*QFt5F-2/QWs5F-1*) has *FpCBF6* as a candidate gene shown to be rapidly up-regulated during CA (Alm et al., 2011).

Conversely, many crops are currently grown in places, where high temperature prevails and field studies have indicated that increase in temperature reduces grain yield of cereals and legumes by 4–14% per 1°C increase (Quarrie et al., 1997). Current projections indicate that both day and night temperatures are likely to increase during this century (Hall and Ziska, 2000) and ideally, heat-resistant cultivars should not only have higher grain yields in hot environments but also similar grain yields as current cultivars in cool atmosphere. Public plant breeding programs have developed heat-resistant cultivars of cowpea, common bean, tomato and Pima cotton that are more productive in hot environments than standard cultivars. Commercial plant breeding companies rarely divulge their methods, but from the available heat-resistant commercial cultivars, it is clear that they have had some success in breeding for heat tolerance during reproductive development in tomato and upland cotton. In the past, very few public or commercial plant-breeding programs gave any emphasis to breeding heat-resistant cultivars. For crops that are sensitive to high temperatures during reproductive development the way forward is to give great emphasis to breeding and finding DNA markers for heat adaptation during flowering.

## Submergence and salinity tolerance

Waterlogging is a major problem for cereal production worldwide, as in sodic environments, soils are affected by seepage from irrigation canals, and excess wetting due to rainfall or floods, especially if it rains after irrigation. Genetic diversity in waterlogging tolerance was reported in various crops, including wheat, barley, maize, and oats (Kerr, 1986), and diverse mechanisms have been associated with tolerance. They are associated with phenology and morphology, nutrition balances, metabolism, including anaerobic catabolism and anoxia tolerance, and post-anoxia damage and recovery (Setter and Waters, 2003). Tolerance of flooding during germination and early seedling growth is essential for direct seeding of rice, both in rainfed and irrigated areas, where even waterlogging is sufficient to cause considerable reduction in crop stand because of their high sensitivity to hypoxia at this stage (Ismail et al., 2009). Substantial genetic variation was recently observed in the ability to germinate and establish in flooded soil. Tolerant genotypes are capable of catabolizing starch reserves in seeds germinating under hypoxia into simple sugars, and use them as substrates to generate energy via anaerobic pathways for the growing embryos (Miro and Ismail, 2013; Septiningsih et al., 2013). Several QTL originating from a few rice landraces were identified, two of them with large effects; on chromosome 9 (*qAG-9-2*) (Angaji et al., 2010) and chromosome 7 (*qAG-7-1*) (Septiningsih et al., 2013). These QTL are being targeted for cloning and for use through MAB, which could eventually result in high yielding rice cultivars for direct seeding in flood-borne areas. Recently, tolerant rice varieties carrying *SUB1* locus became available. *SUB1* is a major QTL on chromosome 9 that has been cloned and the gene responsible for tolerance identified as *SUB1A-1*. This gene encodes an ERF that suppresses ethylene-mediated responses under submergence, and subsequently limits excessive elongation and halts chlorophyll degradation. Both processes are essential to prevent carbohydrate starvation of the submerged plants. These varieties can survive 4–18 days of complete submergence, with yield benefits of 1 to over 3.5 t ha^−1^ (depending on flood duration and floodwater condition), compared to current farmers' varieties, and without any undesirable effects on the features of the original varieties (Singh et al., 2009; Bailey-Serres et al., 2010; Mackill et al., 2012; Ismail et al., 2013). Additional genes are being targeted for submergence tolerance, and once identified they could be combined with *SUB1* for higher tolerance during complete submergence. Further, the progress made in rice could potentially be exploited to improve flood tolerance of other crop species and provide more resilient varieties for current and future flood-affected areas.

Progressive salt accumulation due to excessive irrigation with poor water quality coupled with poor or improper drainage results in high salt levels (Ismail et al., 2007). Numerous studies have characterized responses mediated by salt stress in different plant species and highlighted the complexity of the mechanisms involved (Munns and Tester, 2008). Studies have shown that few major loci and many minor ones were associated with various aspects of salinity tolerance. The best known for rice is *Saltol* on chromosome 1 (Thomson et al., 2010), which possesses a major gene, *OsHKT1;5* (Ren et al., 2005). In wheat, two members of *HKT* gene family (including the wheat *HKT1;5* ortholog) have also been shown to co-localize with major QTL (Byrt et al., 2007). Apparently, many other QTL have been identified in rice and other cereals, and several of them are common across mapping populations. In addition, numerous genes have been identified through functional genomics studies of salt-stress responses, and many of them lead to improved tolerance when they are over- or under-expressed. Some even co-localize with QTL regions, but there has been no further success in using them for breeding tolerant cereal crops or in cloning additional QTL.

Current approaches in this aspect involves using NGS to target major QTL for cloning, and to develop efficient SNP and InDel marker systems to manipulate these loci during MAB. The substantial genetic diversity in the tolerance of salt stress and mechanisms used by various crops to cope with increasing salt concentrations in soil and water provides opportunities to enhance salt-stress tolerance in cereals. However, this will require large investments to dissect and combine the genetic determinants of various traits. Developing such cultivars that are highly tolerant of salt stress is a requisite to cope with the current worsening climatic conditions and to meet the urgent need of producing more food from marginal land and limited water resources.

## Host plant resistance to pathogens and pests

The climatic variables including changes in temperature, rainfall, and atmospheric chemical composition along with predominantly elevated CO_2_ levels would accelerate the reproduction time of many plant pathogens and pests, thereby increasing their infection pressure on crop plants (Boonekamp, 2012). Climate change may also affect the ability of plants to express resistance to pathogens and insects. Experiments conducted by Huang et al. (2009) indicated a 45% increase in leaf lesions in oilseed rape, when the surrounding temperature was increased by 5°C. This finding suggests that the expression and efficacy of R-genes in host plants may be affected where both crop and associated pathogen or pest are affected by climatic variation. This may be influenced by different combinations of selective pressures, and each may respond to these pressures at different rates. Improved understanding on the host-pest/pathogen interactions and knowledge on different effects of climate change is a requisite for the development of climate-resilient crops. To date, research on the impact of climate change on plant diseases has been limited, with many studies focusing on the effects of a single atmospheric constituent or meteorological variable on the host, pathogen, or the interaction of the two, under controlled conditions. Whilst this work is a valuable base to start from, the combined effects of biotic and abiotic stresses must be studied (Ramegowda and Senthil-Kumar, 2015).

Recent advances in genome sequencing and genotyping assays allow for many strategies at the genomics level, which can be developed to understand the impact of climate change on plant diseases. The newly available genome sequences for plants, pathogens and pests provide the resources to study their co-evolution in response to climate change. An understanding of the co-evolution of genes responsible for virulence and resistance will lead to improved plant protection strategies and provide a model to understand plant-pathogen and plant-insect interactions in diverse species. Though it is important to understand the genomics of disease resistance in crop species, and how allelic differences are altering resistance, combining this with studies of CWR or germplasm collections further allows the identification of novel variants. These variants can be used for the introgression of novel resistance genes into cultivars, utilizing the germplasm for breeding and developing new cultivars, or genetic engineering with the advantageous genes. Taken together, it is obvious that the impact of climate change on disease resistance is difficult to predict and is likely to be variable depending on the crop and local environment. However, crop disease is an important factor when considering the impact of climate change on food production and intensive studies applying advanced genomics tools will be required to help ameliorate the impact of climate change on future cropping scenarios in relation to plant disease.

## Genomic engineering tools for targeted mutagenesis by editing genes for adaptation

Plant breeders have been applying mutagenesis to induce genetic variation for increasing crop yield and later, the strategy has been used for improving the adaptability of crop plants. Initially, X-ray radiation was used as a mutagen since it was readily available to researchers (Muller, 1927). Subsequently, gamma-ray radiation has been used to induce point mutations, although chromosomal mutations were also produced (Devreux and Scarascia Mugnozza, 1964). From recent times, chemical mutagenesis is being practiced since they are easy to use, do not require any specialized equipment, and can provide a very high mutation frequency. Compared to radiation methods, chemical mutagens tend to induce SNPs rather than chromosomal mutations. Currently, chemical mutagens, such as Ethyl methanesulfonate (EMS) are being used to induce random mutations into the genome and have become a useful complement to the isolation of nuclear DNA from mutated lines by TILLING (Targeting Induced Local Lesions in Genomes) technology and screening of the M_2_ population at the DNA level using advanced molecular techniques. Single mutations in specific genes for adaptation could be identified by cleavage of mismatched bases formed as a result of repeated melting and reannealing of PCR products amplified from a pool of alleles for the specific gene in a pool of DNA from a set (usually 8) of M_2_ plants (McCallum et al., 2000; Caldwell et al., 2004). NGS can efficiently accelerate the identification of mutations at the whole-genome level. Promotor mutations and mutation in other regulatory elements responsible for the downstream effect can be identified by qPCR, microarray and RNA-seq. Once a mutant allele is identified within gene(s) of interest, those mutations may be linked to a specific phenotype for stress resistance by backcrossing the mutant to the parental line. This TILLING approach is a reverse genetics procedure to associate a mutant allele to its phenotype. Of note, TILLING can also be used for a forward genetic approach by screening phenotypes for adaptation to stresses and then characterize the phenotype using a combination of whole-genome resequencing, linkage maps and microarrays, to gain a broad picture of gene expression changes due to the newly introduced SNPs compared to the original line.

Other molecular tools and resources are now available for genome engineering and reverse genetics experiments in crop plants in order to implement precise manipulation of genetic building blocks and regulatory machinery that underlie yield improvement under stress condition and directly correct harmful mutations by genome editing (Hsu et al., 2014). Targeted genome engineering has emerged as an alternative to classical plant breeding and transgenic methods to improve crop plants and ensure sustainable food production (Belhaj et al., 2013; Osakabe and Osakabe, 2015). Currently, four types of engineered nucleases are used for genome editing: engineered homing endonucleases/meganucleases (EMNs) (Silva et al., 2011), zinc finger nucleases (ZFNs) (Townsend et al., 2009), transcription activator-like effector nucleases (TALENs) (Cermak et al., 2011), and CRISPR (clustered regularly interspaced short palindromic repeats)/Cas (CRISPR-associated)9 (Cong et al., 2013; Mali et al., 2013).

Sequence-specific nucleases (SSN) enable precise genome engineering by introducing DNA double-strand breaks (DSB) that subsequently trigger endogenous DNA repair by non-homologous end joining (NHEJ) or homology directed repair (HDR) recombination mechanisms in different species. Site-directed mutagenesis mediated via NHEJ can be achieved while HDR cause directed gene knock-in/correction at specific locations in the genome (HDR uses a DNA template to replace the DNA sequence at the break point). NHEJ functions throughout the entire cell cycle whereas HR is restricted to late S/G2 phases in the cell cycle. Therefore, NHEJ is the major DSB repair pathway in eukaryotes. Belhaj et al. (2013) and Osakabe and Osakabe (2015) display a clear illustration of genome editing assays in model (*Arabidopsis thaliana* and *Nicotiana benthamiana*) and crop (*Oryza sativa, Triticum aestivum* and *Sorghum bicolor*) plant species. These SSN effects generate targeted genome modifications including mutations, insertions, replacements and chromosome rearrangements and have been induced in a variety of important crops, such as rice, maize, wheat, barley, and soybean. Each technology has advantages and disadvantages with regard to cost, ease of construction, efficiency of targeting, and specificity (Chen and Gao, 2014; Gao, 2015). Major advantages of ZFNs are related to the acceptance of the technology as no transgenic is produced because viral vectors have been used for expressing transiently the nuclease, which do not integrate into the genome. However, it has disadvantages such as difficulties to design the experiments, limited number of target sites, and the regeneration of juvenile and chimeric mutated plants when custom-designed nucleases have been delivered in tree explants.

CRISPR was first discovered as an immune system of prokaryotes, which subsequently became a powerful tool for genome editing in eukaryotes (Gaj et al., 2013). It has emerged as an alternative to classical plant breeding and transgenic methods to improve crop plants. Plant transformation and co-expression of the Cas9 with a chimeric guide-RNA (gRNA) targeting a GN19NGG motif in the gene of interest, results in a double-strand non-self DNA cleavage on both strands at a specific site near the protospacer adjacent motif (PAM) (Gasiunas et al., 2012; Jinek et al., 2012). The Cas9 from *Streptococcus pyogenes* (SpCas9) recognizes 5′-NGG-3′ as the PAM sequence (Hsu et al., 2013). PAM plays an important role in target binding and cleavage by the Cas9–gRNA complex (Lei et al., 2014). CRISPR/Cas9 has greater number of advantages, including the straightforward construct design and assembly and the achievement of high mutation rates, matching or exceeding those obtained with ZFNs and TALENs. Only 20 nucleotides in the gRNA need to be modified to recognize a different target making unnecessary the sophisticated protein engineering for each target that is crucial for the other SSN approaches.

So far, the CRISPR-Cas9 technology has been applied in *A. thaliana* (Feng et al., 2013, 2014; Jiang et al., 2013; Li et al., 2013; Mao et al., 2013) *Nicotiana benthamiana* (Jiang et al., 2013; Li et al., 2013; Nekrasov et al., 2013), *Oryza sativa* (Feng et al., 2013; Jiang et al., 2013; Mao et al., 2013; Miao et al., 2013; Shan et al., 2013; Xie and Yang, 2013; Zhang et al., 2014a), *Solanum lycopersicum* (Brooks et al., 2014), *Sorghum bicolor* (Jiang et al., 2013), *Triticum aestivum* (Wang et al., 2014), *Citrus sinensis* (Jia and Wang, 2014), and *Populus tremula* x *alba* (Zhou et al., 2015). Genes controlling traits of importance for adaptation have also been edited by CRISPR-Cas9 technology. gRNAs were designed to target three specific sites of the rice *OsMPK5* gene which encodes a stress-responsive rice mitogen-activated protein kinase and the targeted mutation of *OsMPK5* enhanced rice disease resistance (Xie and Yang, 2013). Transgenic wheat plants carrying mutations in *TaMLO-A1* allele were susceptible to powdery mildew diseases (Wang et al., 2014). The bacterial blight susceptibility genes, *OsSWEET14* and *OsSWEET11*, were targeted for mutation at the promoter region in Arabidopsis, tobacco, sorghum and rice (Jiang et al., 2013). High CRISPR-Cas9 mutational efficiency was achieved for three 4-coumarate:CoA ligase (4CL) genes, *4CL1, 4CL2* and *4CL5*, associated with lignin and flavonoid biosynthesis in *Populus tremula* x *alba* (Zhou et al., 2015).

Moreover, accelerated breeding of crop plants carrying targeted gene mutation(s) without foreign DNA is possible using CRISPR genome editing. In fact, although transgene Cas9 and selectable marker integration is hemizygous, CRISPR editing at the target loci is biallelic. Therefore, in autogamous plants, self-fertilization of T_1_ plants will provide 25% of the T_2_ plants without the transgene but maintaining the edited gene in homozygosity. In self-incompatible or dioecius perennial woody trees, biparental hemizygous Cas9/sgRNA transformation and biallelic-edited gene can be produced. Controlled crosses between male and female primary transformants with confirmed biallelic mutations should produce transgene-free, biallelic mutants in 25% of the progeny (Zhou et al., 2015). Taken together, genome engineering for targeted mutagenesis by editing genes serves as a potential strategy for generating elite cultivars of crop plants with durable climate resilience.

## Concluding remarks

Global climate change is predicted to impose a severe threat to agricultural productivity worldwide, and thereby challenge food security and nutritional security. Technological advances, particularly transgene-based and molecular breeding technologies have facilitated the development of elite genotypes with durable adaptation to climate change. Genomics-assisted breeding in particular is predicted to playing a significant role in the development of climate change resilient crops. Excellent model organisms for climate change such as foxtail millet and green foxtail (for C_4_ photosynthesis), *Brachypodium* (grass model) have been identified for deciphering traits that need to be decoded and introgressed in the crop plants. Advances in DNA sequencing technologies and the sequencing of CWR, along with advanced genomics tools will expedite the identification of novel genes and key regulatory regions of stress tolerance toward the development of new cultivars with durable resistance. Although, the impact of climate change on crop's resistance is difficult to predict and is likely to be variable depending on the crop and environment, genomics-assisted breeding could contribute significantly to reduce the impact of climate change on future cropping scenarios.

## Author contributions

All the authors contributed equally to the manuscript. CK prepared the outline of the contents, identified the authors from the members and advisors of the International Climate Resilient Crop Genomics Consortium (www.icrcgc.org), and coordinated the drafting of the manuscript. RH, DE and RS assisted in editing. CK, MP and MM revised the manuscript and prepared the final version.

### Conflict of interest statement

The authors declare that the research was conducted in the absence of any commercial or financial relationships that could be construed as a potential conflict of interest.
